# An Account of a Remarkable Fracture of the Scull, by a Pistol-Ball, That Entered the Cranium at the Right Temple and Was Successfully Extracted

**Published:** 1783

**Authors:** 


II. *An account of a remarkable Fracture of the fcull, by a piftol-ball, that entered the cranium at the right temple and was fuccefsfully extracted. By Mr.* Cook, *Surgeon at Barking in Effex. Communicated by Dr.* Ofborn, *Phyfician in London.*

IN the month of Auguft laft a blackfmith, of the parifh of Dagenham in Effex, having taken the defperate refolution to fhoot himfelf, applied a loaded horfe-piftol to his right temple. The ball entered the cranium, obliquely, at the os frontis, clofe to the os fphenoides, paffed along the infide of the cranium to the os frontis about two two inches above the futura tranfverfalis, and feparated an oblong piece of the whole fubftance of the cranium three inches in circumference; the refiftance from which threw the ball back again. Upon examining him about an hour after the accident I found a hard fwelling on the forehead, which I fuppofed to be the ball; but, on cutting through the integuments, which were not the leaft injured externally, I found the piece of cranium as above defcribed intirely feparated. This portion of bone was eafily removed; but I could not then find the ball. I therefore dreffed the depending opening I had made above the nofe, and left him till next day, when my affiftant, who faw him firft, found the ball fuperficially lodged among the fractured bones, and eafily extracted it. The wound was dreffed in the ufual manner, and for feveral days he was almoft infenfible of any thing that was faid or done to him; but had none of thofe violent fymptoms which often attend a fractured cranium. In about ten days his fenfes became tolerably perfect, his fever abated, and his wound had a very good afpect. Several fmall exfoliations came away at different times, and in feven weeks the wound was perfectly healed. The veffels leading to his right eye were deftroyed in

 the the first instance, and the muscle of the eye-lid lost its power of contracting, so that his eye remained uncovered as well as blind. The ball way very much cut and indented by the resistance from the bone, my patient having assured me, that it was perfectly smooth when he charged the pistol with it.

He is now able to work at his business, except striking upon the anvil, which shakes the new bone too much for him to bear at present.

*Barking in Essex,*

*Dec.* 12, 1782.

